# Effect of structured training programme on the knowledge and behaviors of breast and cervical cancer screening among the female teachers in Turkey

**DOI:** 10.1186/s12905-017-0478-8

**Published:** 2017-12-07

**Authors:** Ayla Bayık Temel, Şafak Dağhan, Şenay Kaymakçı, Renginar Öztürk Dönmez, Zeynep Arabacı

**Affiliations:** 1 Department of Public Health Nursing, Ege University Nursing Faculty, 35100 İzmir, Turkey; 2 Department of Nursing, Near East University, Lefkoşa, Turkish Republic of Northern Cyprus; 3 Department of Nursing, Kastamonu Universiy, Tosya Vocational School, Kastamonu, Turkey

**Keywords:** Breast and cervical cancer, Knowledge, Attitude, Behavior, Teacher

## Abstract

**Background:**

Breast cancer and cervical cancer are the most common cancers among women in the world. Many studies on the early detection of cancer have been conducted among women worldwide, but few studies have been performed in the world on female teachers regarding breast self-examination (BSE), mammography (MMG) and Pap smear test (PST). As teachers interact with students, this could play an important role in health education and in developing healthy behavior such as cancer screening. The main objective of this study was to evaluate the effect of a structured teaching program on breast and cervical cancer screening on the knowledge and practice of teachers. The other objective was to encourage teachers to transfer this knowledge to the women who attended their courses.

**Methods:**

Semi –experimental designs with pre-intervention, post-intervention and six month follow-up tests were used in this study. The data were collected from 37 volunteer teachers and their 64 volunteer students with a sociodemographic form, a questionnaire form for breast and cervical cancer, and a Transtheoretical Model of behavior change for BSE, MMG and PST. Behavior of the teachers related to BSE, MMG, PST was evaluated in pre-training and in the first, third and sixth months post-training, and the behavior of the students was evaluated with point follow-up in the sixth month.

**Results:**

In post-training, it was determined that the teachers’ knowledge of breast cancer increased from 11.70 ± 2.80 to 14.81 ± 3.22 and their knowledge of cervical cancer increased from 7.75 ± 5.60 to 17.68 ± 3.79. For BSE behavior, 47.8% of teachers were in the action and maintenance stage in pre-training, but this ratio was 81.1% in the sixth month post-training. For MMG behavior, all of the teachers were in the precontemplation stage in pre-training, and 38.9% of them were in the action and maintenance stage in the sixth month post-training. For PST, while 24.3% were in the action and maintenance stage in pre-training, this ratio was 45.9% in the sixth month post-training.

**Conclusion:**

It was determined that the behavior change for BSE, MMG, PST was positive. Similarly, knowledge transfer from teachers to students was also effective.

**Electronic supplementary material:**

The online version of this article (10.1186/s12905-017-0478-8) contains supplementary material, which is available to authorized users.

## Background

Breast cancer (BC) is the most common cancer among women in the world. Epidemiological data indicate that one in three women in the world experience cancer [1]. BC accounts for 25% of all types of cancer globally and 40.6% of all cancer cases among women in Turkey in 2009 [1, 2]. Furthermore, cervical cancer (CC) is the third most common cancer in developed countries and the second most common cancer in developing countries among women after breast cancer. CC accounts for 12% of all types of cancer globally [1]. In Turkey, CC is the 10th most frequent (4.5%) cancer among all cancers of women [2].

Early detection, early referral and prompt treatment would be helpful to reduce its mortality. Breast self-examination (BSE), mammography (MMG) and clinical breast examination (CBE) are believed to be appropriate and effective methods for ensuring early detection of breast cancer [3, 4]. In studies in community samples of diverse groups of women, the rates for performing BSE ranged from 10.2% to 54.8% in different countries. Women in developed countries perform BSE more frequently and have higher level of knowledge compared to those in developing countries [4–9]. The results of epidemiological studies on BSE in Turkey have shown that the percentage of women who knew how to perform BSE range from a low 9.9% to a high 45.1% [10–14].

Population-based Pap smear test (PST) programmes for CC have shown the effectiveness of screening in reducing mortality [15, 16], while CC screening has become a success story in cancer prevention in developed countries, this cannot be said for developing countries, which still bear the burden of this preven**t**able malignancy. Despite the fact that about 80% of all cervical cancers occur in developing countries, in general, developing countries have lower PST coverage (2.6%–19%) compared to developed countries (63%–99%) [9, 17–21]. In Turkey, undergoing the PST ranges from 11.8% to 68.5% among women [22–24]. The Turkish Ministry of Health has issued guidelines regarding CC screening and recommended that all women aged 35–40 years should perform at least one PST in 5-year intervals and screening would be finalized for 65-year-old women whose last tests are negative [25].

The Transtheoretical Model (TTM) is applied successfully to motivational and cognitive processes of behavioral change with respect to early diagnosis screening such as BSE, MMG and PST to assist in the development of behavioral change strategies more appropriately matched to a women’s readiness to act [26–29]. Many researchers have also applied the TTM to identify and promote breast and cervical cancer screening [28–31]. The TTM proposes that individual moves through a temporal sequence of several stages of behavioral change [26–31].

Many studies on BSE, MMG and PST practice have been conducted among women worldwide and in Turkey in general and in certain groups of women such as health workers, academicians at college or university level, factory workers, and female students at college or university level [5, 8, 18, 24, 32]. However, only a few studies have been performed on female teachers regarding BSE and PST in the world [5, 6, 8, 33–35]. In our country, many studies have focused on BSE and MMG screening among teachers; however, there is no research on PST screening among them [11, 12]. As teachers interact with students, it is vital for them to serve as role models of character by practising BSE and applying for PST as well as becoming the educational role model by teaching and disseminating reproductive health knowledge [6, 7, 33, 35]. The difference distinguishing this study from others in this field is that it comprises the transfer of information regarding educational programmes directed towards breast cancer and cervical cancer from researchers to teachers, and from teachers to students, and evaluation of the results. Furthermore, the main aim of this study was to evaluate the effect of breast and cervical cancer screening-structured teaching programme on the knowledge and behaviors of female teachers in a public training centre. The other objective was to encourage teachers to teach and share the knowledge and skills with the women who attend their classes and courses in public training centres, and also to evaluate the diffuseness of the teachers’ training efforts on the students’ BSE, MMG and PST behaviors.

## Methods

### Design, study setting and population

The semi*-*experimental designs with pre-intervention, post-intervention and six-month follow-ups period was used in the study.

Public training centers are institutions that are opened in different periods providing regular and organized training courses to adults [36]. At the time of the study, the number of teachers continuing at the public training center was 37. The study was conducted with 37 volunteering female teachers (participation rate = 100.0%) and their 64 volunteering students between December 2009 and June 2010. The sufficiency of the size of the sample was calculated following determination of the extent of the relationship of the effect size regarding BSE, MMG and PST. Accordingly, taking the effect size for BSE as 0.66, the test strength as %80 and the level of significance as α = 0.05, the size of the sample of this study was determined as 32. Taking the effect size for PST as 0.92, the test strength as %80 and the level of significance as α = 0.05, the size of the sample of this study was determined as 14. Taking the effect size for MMG as 1.55, the test strength as %80 and the level of significance as α = 0.05, the size of the sample of this study was determined as 8 [37]. The sample size (*n* = 37) has been determined to be sufficient for all parameters.

### Intervention

After having obtained the approval of the directorate of the public training center, the first stage of the project began with a conference for awareness of all the teachers on breast cancer and cervical cancer at the beginning of the term (September–December, 2009). Information regarding the importance of breast and cervical cancer prevention was given and attracted their attention and tried to raise awareness. At the end of the conference, the researchers discussed the objectives of the project and the training program and the responsibilities of the participants. Among the teachers, 37 gave consent to participate as a volunteer in the study. On the same day, the teachers were divided into two subgroups; each group consisted of 12–13 participants, and they were asked to fill the self-administered questionnaire (pretest) in order to assess their knowledge level related to BC, BSE, CC, PST, information sources, barriers and attitudes, and the status of the stages of change.

*Two weeks later,* each group was given two theoretical and one practical session (each session taking 60 min) on anatomy, physiology of reproductive system, BC, CC, symptoms, risk factors, early detection, prevention, treatment methods, BSE, MMG and PST. The training sessions were carried out in the form of lectures, group discussions, demonstrations, and through use of models and self-examination. After the researchers demonstrated how to practice BSE, each participant was encouraged for palpation practice on a *breast* model to teach fingers how to feel several lumps*.* An educational video film prepared by the Association of Breast Cancer Crusade (in Turkish: Meme Kanseri ile Savaşım Derneği) on BSE practice was displayed and a CD, documents, brochure, and magnet with a message (***Don’t be afraid of cancer, learn how to prevent and share your knowledge***) were given out to the participants for self learning and to reinforce their memories. One week later, another theoretical and practical session was organized and an observation checklist was used to evaluate the improvement in the proficiency of BSE practice. Three weeks later in February 2010, a post-test was implemented. The pre-test and post-test were compared via pseudonyms.

In the third stage of the project, the teachers’ behaviors related to BSE, applying for MMG and PST and their status of the stages of change were checked once every two months through telephone interviews in the six-month (February, April, July) follow-up time period. If a participant reported that she had applied for PST and MMG at least once, only the BSE behaviors were evaluated. At the end of the training program, the teachers transferred the knowledge and skills to their volunteer students (*n* = 64) through the lecture method and using video film. The behaviors of the students related to BSE, PST and MMG were evaluated with point follow-up before the training and after training at the 6th month.

### Measurements/instruments

The data were collected with the following forms.

#### Sociodemographic form

The data were collected via the questionnaire form including 22 questions on the teachers’ demographic gynaecological characteristics and history of cancer.

#### Questionnaire form for BC, BSE, MMG, CC and PST

This questionnaire was developed through literature search [3, 16]. This questionnaire consisted of two parts. The first part included the knowledge questions for BC, MMG, BSE and the second part included the knowledge questions for CC and PST. Each question was answered as correct and false. A score of one (1) was awarded for each correct answer and zero (0) for each wrong answer.

The knowledge of the teachers regarding BC, BSE and MMG was assessed with 21 questions (Min = 0, Max = 21). A score of 0–7 points obtained from this questionnaire was determined as low knowledge level, 8–15 points as mid-level knowledge, and a score of 16–21 points was determined as high level knowledge. The knowledge of the teachers regarding CC and PST was assessed with 26 questions (Min = 0, Max = 26). A score of 0–8 points obtained from this form was evaluated as low knowledge level; a score of 9–17 points as mid-level point and a score of 18–26 points was evaluated as high level knowledge.

In order to test the reliability, the KR-20 coefficient was calculated. Accordingly, the KR-20 alpha coefficient as 0.670 was determined as an acceptable measure [38]. In order to obtain an expert opinion with regard to the context of the validity of the scope of the questionnaire forms, the forms have been evaluated by the members of the Association of Breast Cancer Crusade and the final version of the questionnaire form has been determined through minor changes made according to recommendations (for example, not carrying out BSE after cessation of menstruation, but on the 5th – 7th day after start of the menstruation, questioning the age at the time of marriage instead of the age at first sexual experience). Hypothesis testing was performed in this study to test the construct validity of the data collection form. The verification of the hypothesis shows that the measurement is valid [39]. The hypothesis is “There is a linear relationship among realizing early diagnosis behaviors as knowledge on cancer and the early diagnosis methods of cancer increases.” Accordingly, the intermediate level of positive and statistically significant correlation (*r* = 0.363, *p* = 0.030) was found between the knowledge score of the teachers on cervical cancer and early diagnosis methods before the training and PST making behaviour (action stage). The hypothesis has been verified. The intermediate level of negative and statistically significant correlation (*r* = −0.330, *p* = 0.046) was determined between the teachers’ knowledge score on breast cancer and early diagnosis methods before the training and BSE making behaviour (action stage). The hypothesis has not been verified.

Consequently, while the validity and reliability of the cervical cancer and early diagnosis methods questionnaire form were ensured in the context of the data collection form, the reliability and partial validity of the breast cancer and early diagnosis methods questionnaire form were ensured in the study.

#### Stages of change

The behaviors of teachers who had carried out BSE, MMG and PST before training (time 1), after educational first (time 2), third (time 3) and sixth (time 4) month follow-ups and the students to whom knowledge was transferred by teachers at the sixth month follow-ups were assessed according to changing phases form developed by Prochaska and Diclemente (1983–1992) [40]. The stages of change for BSE, MMG and PST have been defined in Table 1 [26–28, 30, 31].

**Table 1:** Stages of Change according to BSE, MMG and PST

Stages of change	BSE	PST or MMG
Precontemplation stage	Women in this stage responded that they were not currently performing BSE montly and were not seriously considering performing BSE within the next 6 months	Women never had a mammogram or PST, and nor planning to get one within the next 6 months
Contemplation stage	Women in this were not currently performing BSE montly but were seriously considering performing initiating montly BSE within the next 6 months	Women never had a mammogram or pap test but intends to get one within the next 6 months to one year
Preparation stage	Women in this stage were not currently performing BSE montly but they intended to perform BSE as early as the next month	–
Action stage	Women in this stage were currently performing BSE monthly	Women had mammogram or pap test on schedule and intends to get another on a time frame that will keep the women on Schedule
Maintenance stage	Women in this stage were currently performing BSE for at least 6 months	–
Relapse	–	Women had one or more mammograms or pap tests in the past but is now off schedule and does not plan to have a mammograms or pap tests within future.

### Statistical analysis

The data were analysed using the SPSS 16.0 statistical software. The suitability for normal distribution was tested using the Kolmogorov-Smirnov analysis. The validity and reliability was tested using KR-20 and Spearman’s correlation analysis. The dependent t test, Wilcoxon and the McNemar test were used to assess the differences in the pre-test and post-test scores. A p level of <0.05 was considered statistically significant. For the repeated measures, the Friedman test and Wilcoxon sign rank test were used to examine and compare the time periods. The effect size was calculated for the difference of knowledge scores and stages of changes in terms of breast and cervical cancer. The acceptable effect size was determined as >0.05 [41].

## Results

### Descriptive information of teachers and health histories

The mean age of the teachers was 39.91(SD = 10.12) (Range = 22–57), 48.6% of them were above 40 years of age, 81.1% had a bachelor’s degree, 62.2% were married, all of them had health insurance, and 70.3% did not smoke. The mean age of the students was 38.58 (SD = 10.83) (Range = 21–61), 25.0% (*n* = 16) of them were above 40 years of age and 75.0% were married.

The mean menstruation age of the teachers was 13.36 years (SD = 1.07), the mean age of marriage is 23.88 years (SD = 4.22), the mean number of pregnancies was 2.19 (SD = 0.92), the mean age at first delivery was 25.38 years (SD = 4.89). Of the teachers, 35.3% were in menopause and the mean age for menopause was 46.41 years (SD = 4.89). Of the teachers, 83.3% had gained weight after menopause, and 37.0% received hormone replacement therapy (HRT). The mean duration for receiving HRT was 4.71 years (SD = 3.72) (Min = 1, Max = 12). 3.7% of the teachers had history of breast cancer and 12.4% had family history for breast cancer. The rate of women experiencing the complaint of bleeding following sexual intercourse was 3.3%.

### Knowledge of the teachers regarding to breast and cervical cancer

Table 2 shows the knowledge of pre-training and post-traning regarding the risk factors for breast cancer and the early diagnosis methods. The mean knowledge score of the teachers regarding breast cancer before the training programme was 11.70 ± 2.80 (Min = 9, Max = 20) and after training, it was 14.81 ± 3.22 (Min = 9, Max = 20). It was determined that with regard to the knowledge score regarding breast cancer, the average score for both the pre-test (Kolmogorov-Smirnov Z = 0.812, *p* = 0.525) and the post test scores (Kolmogorov-Smirnov Z = 0.759, *p* = 0.612) demonstrated a normal distribution. A statistically significant difference was determined between the pre-test and the post-test (*t* = 5.78, *p* = 0.01). The calculated effect size regarding breast cancer and BSE knowledge scores was 1.03. The rates of correct knowledge regarding the risk factors before the training was 18.9% and 89.2% and the rate of change was between 62.2% - 97.3% at the end of the training. The risk factors that were less known before the training and more known after the training were determined as “being a woman”, “early menstruation or late menopause”, “to have the first delivery after the age of 30 years or never having breastfed”, “using hormone replacement therapy (HRT)” and “being obese” (*p* < 0.05). The true knowledge of these factors increased by 35.2%–46.0% after the training. Most of the teachers had answered the questions regarding BSE application techniques (81.1%) and frequency of application (73.0%) correctly before the training. At the end of the training, while the same questions were answered correctly, the highest correct response that was higher than the pre-training was the BSE performing time in questions regarding menstruating and menopausal women (*p* < 0.05). At the end of the training, the correct answering rates were increased as 16.2%–24.3% at the end of the training (*p* < 0.05).

**Table 2:** The knowledge of pre-training and post-traning regarding breast cancer risk factors and early diagnosis methods (*n* = 37)

Variables	Pre-test		Post-test		Difference (%)	Significance test	
	*n*	%	*n*	%		x^2^	*p*
Breast cancer risk factors							
Being a women							
True	12	32.4	25	67.6	35.2	3.153	0.002
False	25	67.6	12	32.4			
Being over 50 years old							
True	18	48.6	23	62.2	13.6	1.387	0.267
False	19	51.4	14	37.8			
Have a family history of breast cancers							
True	33	89.2	36	97.3	8.1	1.342	0.375
False	4	10.8	1	2.7			
Started menstruating younger than age 12 or go through menopause older than 55							
True	8	21.6	25	67.6	46.0	4.123	0.000
False	29	78.4	12	32.4			
Have the first delivery after the age of 30 years or, never having breastfed							
True	8	21.6	25	67.6	46.0	4.123	0.000
False	29	78.4	12	32.4			
Using hormone replacement therapy							
True	21	56.8	28	75.7	18.9	2.309	0.039
False	16	43.2	9	24.3			
Being obese							
True	7	18.9	23	62.2	43.3	3.771	0.000
False	30	81.1	14	37.8			
Application tecnique of BSE							
When should a women begin BSE?							
True	23	62.2	27	73.0	10.8	1.160	0.254
False	14	37.8	10	27.0			
How often should be performed?							
True	27	73.0	33	89.2	16.2	1.970	0.047
False	10	27.0	4	10.8			
When should a women with menstruation do BSE?							
True	6	16.2	15	40.5	24.3	2.700	0.010
False	31	83.8	22	59.5			
When should a women with irregular menstruation do BSE?							
True	26	70.3	33	89.2	18.9	2.021	0.041
False	11	29.7	4	10.8			
What is the correct BSE tecnique?							
True	29	78.4	32	86.5	8.1	0.702	0.487
False	8	21.6	5	13.5			

Table 3 demonstrates the knowledge of pre-training and post-traning regarding cervical cancer risk factors and early diagnosis methods. The mean score of knowledge of the teachers in terms of CC risk factors and its early diagnosis was 7.75 ± 5.60 (Min = 1, Max = 18) before the training and 17.68 ± 3.79 (Min = 12, Max = 25) after the training. While the pre-test score regarding cervical cancer did not demonstrate a normal distribution pattern (Kolmogorov-Smirnov Z = 2.877, *p* < 0.05), the score of the post test was determined to show a normal distribution (Kolmogorov-Smirnov Z = 0.678, *p* > 0.05). A statistically significant difference was determined between the pre-test and the post-test scores (Z = 4.886, *p* < 0.05). The effect size value calculated for cervical cancer and the PST knowledge score was 2.11. The teachers had answered the questions related to CC risk factors correctly at a rate of 19.0%–54.1% before the training and 37.8%–89.2% at the end of the training. The correct knowledge of the questions after training had increased by 2.7%–46.0%. The CC risk factors that were less known before the training and more known after the training were determined as “early age at first sexual intercourse”, “number of full-time pregnancies” and “smoking” (*p* < 0.05).

**Table 3:** The knowledge of pre-training and post-traning regarding cervical cancer risk factors and early diagnosis methods (*n* = 37)

Variables	Pre-test		Post-test		Difference (%)	Significance test	
	*n*	%	*n*	%		x^2^	*p*
Cervical cancer risk factors							
Women who have early age sexual intercourse							
True	14	37.8	31	83.8	46.0	3.710	0.000
False	23	62.2	6	16.2			
Have had four or more full-term pregnancies							
True	7	19.0	19	51.4	32.4	3.207	0.002
False	30	81.0	18	48.6			
Not to pay attention to hygiene rules							
True	17	45.9	21	56.8	10.9	0.943	0.481
False	20	54.1	16	43.2			
Having any disease in the genitals of their partner							
True	13	35.1	14	37.8	2.7	0.302	0.763
False	24	64.9	23	62.2			
Have a smoke							
True	20	54.1	33	89.2	35.1	3.606	0.000
False	17	45.9	4	10.8			
Have a sexually transmitted diseases							
True	16	43.2	17	45.9	2.7	1.155	0.248
False	21	56.8	20	54.1			
Application of Pap test							
What should be done to protect the cervical cancer?							
True	9	24.3	28	75.7	51.4	1.434	0.160
False	28	75.7	9	24.3			
Knowledge Implementation period of HPV vaccines							
True	2	5.4	8	21.6	16.2	2.960	0.005
False	35	94.6	29	78.4			
Knowledge of why colonoscopy is implementation							
True	35	94.6	37	100.0	5.4	0.326	0.574
False	2	5.4	0	0.0			
Knowledge what should bedone before Pap test							
True	8	21.6	11	29.7	8.1	3.718	0.001
False	26	78.4	26	70.3			
How often should pap smear test be performed?							
True	1	2.7	5	13.5	10.8	7.063	0.000
False	36	97.3	32	86.5			

The teachers mostly knew about the aim of colposcopy before and after the training regarding the PST (94.6%–100.0%). A difference was found between the correct response rates of the “implementation period of Human Papilloma Virus (HPV) vaccines”, “to know what should be done before the PST” and “the frequency of undergoing the PST” questions before and after the training (*p* < 0.05). The correct knowledge of these questions regarding PST had increased by 5.4%–51.4% after training (Table 3).

### Performing BSE, MMG and the PST behaviors of the teachers

It was determined that 48.6% of the teachers had carried out BSE in time 1. Forgetting (41.7%), not knowing how to do it (33.3%), fear of finding a mass (16.7%), and consideration of BSE is not effective (8.3%), were determined as the reasons for not performing BSE. None of the teachers between the ages of 40–57, who should have undergone MMG (48.6%) were determined not to have undergone MMG, and 70.3% of all the teachers had not undergone PST. Unwillingness to undergo PST (36.4%), suggesting it as unnecessary (36.4%) and not allocating time for this procedure (27.3%) were determined as the reasons for not undergoing PST.

The appropriateness of the distribution of the data regarding the BSE, PST and the MMG behaviors of the teachers for each follow-up (time1-time4) was tested using the Kolmogorov-Smirnov analysis. Accordingly, the data of all follow-ups regarding the BSE and PST behaviors were determined not to be suitable for a normal distribution (Z_BSE_ = 1.804, 1.454, 1.447, 2.661; *p* < 0.05 Z_PST_ = 1.631, 1.631, 1.354, 1.671; p < 0.05). However, in the data belonging to the MMG behavior, time 1 and time 2 were found not to match the normal distribution (Z = 2.127, 2.001; *p* < 0.05), and time 3 and time 4 were determined to match the normal distribution (Z = 1.102, 1.154; *p* > 0.05). The BSE, MMG and PST behaviors of the teachers were assessed as the stages of changes before time1, time 2, time 3 and time 4 follow-up periods. The results have been presented in Table 4. With regard to performing BSE, while 48.7% of the teachers were in the action and maintenance stage in time 1, this rate was 81.1% in time 4. The difference between the stages of change was statistically significant (x^2^ = 8.469, *p* < 0.05). The stages of change were significant when time 1 was compared with time 2 and 4 in the advanced analysis (Z = −2.004, −2.270; p < 0.05). A medium level effect size was determined between the follow-ups, in terms of performing BSE (d = 0.66). In the assessment of the MMG behavior, while all of the teachers were in the stage of contemplation in time 1, 38.9% of the teachers were in the action and maintenance stage in time 4 (x^2^ = 44.769, *p* < 0.01). The stages of change were significant when Time 1 was compared with time 3 and 4 in the advanced analysis (Z = −3.525, −3.624; *p* < 0.01). The effect size of the mammography behavior change between the follow-ups was determined as 1.55. With regard to undergoing PST, 24.3% of the teachers were in the action and maintenance stage in time 1, this rate was 45.9% in time 4. The difference between the stages was statistically significant (x^2^ = 31.022, *p* < 0.05). The stages of change were significant when Time 1 was compared with time 2, 3 and 4 in the advanced analysis (Z = −3.525, −3.624; *p* < 0.01). The calculated effect size between the follow-ups with regard to undergoing PST was 0.92.

**Table 4:** BSE, MMG and PST Behaviors of the teachers according to follow-ups (*n* = 37)

Stages of change	Pre-training		Post-training						Significance test^a^	
	Time 1		Time 2		Time 3		Time 4			
	*n*	%	*n*	%	*n*	%	*n*	%	x^2^	*p* ^b^
Stages of change related BSE (*n* = 37)										
Contemplation	6	16.2	3	8.1	3	8.1	3	8.1	15.211	0.002
Preparation	13	35.2	9	24.3	5	13.5	4	10.8		
Action	3	8.1	9	24.3	13	35.1	3	8.1		
Maintanance	15	40.6	16	43.2	16	43.2	27	73.0		
Stages of change related MMG (*n* = 18										
Precontemplation	18	100.0	15	83.3	3	16.7	2	11.1	44.769	0.000
Contemplation	0	0.0	3	16.7	9	50.0	9	50.0		
Action and Maintenance	0	0.0	0	0.0	6	33.3	7	38.9		
Stages of change related PST (*n* = 37)										
Precontemplation	20	54.1	10	27.0	4	10.8	4	10.8	31.022	0.000
Contemplation	6	16.2	12	32.4	13	35.1	13	35.1		
Action and Maintenance	9	24.3	13	35.1	17	45.9	17	45.9		
Relapse	2	5.4	2	5.4	3	8.1	3	8.1		

^a^Friedman analyses with Bonferroni correction

^b^Cronbach Alpha used as .016 (α/3 = .016)

### Performing of the BSE, MMG and PST behaviors of the students

Performing of the BSE, MMG and PST behaviors of the students was evaluated with point follow-up before the knowledge transfer by teachers and after the 6th month. Before the knowledge transfer with regard to performing of BSE, 43.7% of the students were in the pre- contemplation stage, 20.3% were in contemplation stage, 23.5% were in the preparation stage and 12.5% were in the action stage. At the 6th month point follow-up; 6.2% were in the pre-contemplation, 37.5% were in the contemplation, 20.3% were in the preparation stage, 23.5% were in the taking action stage, and 12.5% were in the maintenance stage (x^2^ = 17.000, *p* < 0.05). Of the students over 40 years of age, 81.2% of those for whom undergoing MMG was required were in the action and maintenance stage before the knowledge transfer, and all of them passed to this stage at the 6th month point follow-up (x^2^ = 1.861, *p* > 0.05).

When the behavior of undergoing PST was examined, prior to the knowledge transfer, 63.5% of the students were in pre- contemplation stage, 33.3% were in the action and maintenance stage, and 3.2% were in the relapse stage. In the point follow-up, 30.2% of the students were in pre-contemplation stage, 66.6% were in the action and maintenance stage, and 3.2% were in the relapse stage (x^2^ = 4.690, *p* < 0.05).

## Discussion

In this research, the main aim was to evaluate the effect of breast self-examination and cervical cancer screening-structured teaching programme on knowledge, attitude and practice of female teachers and to encourage teachers to teach and share this knowledge and skills with the women who attended their classes and courses in the public training centres, and also to evaluate the diffuseness of the teachers’ training efforts on the students’ behaviors towards BSE and PST.

### Knowledge of teachers in terms of breast and cervical cancer

Knowledge is the first necessary step for the development of behavior [42, 32]. In this study, the knowledge scores of the teachers regarding breast and cervical cancer before the education was determined as medium-low level. Finding a higher knowledge of teachers regarding breast cancer may be attributed to breast cancer public awareness campaigns being carried out more frequently than cervical cancer. Similarly, a medium-low level knowledge of the teachers has been determined regarding breast and cervical cancer in various studies conducted in various countries on the subject [6, 11, 12, 33–35]. There was an increase in the knowledge level of the teachers determined at the end of the training on breast and cervical cancer, although their knowledge level scores before the training for cervical cancer was found to be lower than breast cancer knowledge level scores, and a higher increase of cervical cancer knowledge level score was determined at the end of the training. The increases in the knowledge for both cancers suggest the effectiveness of the given training.

After the training, an increase in the teachers’ knowledge about breast cancer risk factors was determined, and parallel to the study results, age, heredity, delivery at advanced age, gender and breast feeding were determined as the most commonly known risk factors in similar studies about the subject [5, 8, 12, 32].

The knowledge of the teachers before the training about the age at which BSE should be initiated, the frequency, methods used and the BSE timing for the menopausal women (62.2%–81.1%) was highly sufficient; however, it was determined that they knew less about the time for performing BSE in menstruating women (16.2%). After the training, a significant increase in the BSE frequency and knowing the timing of BSE for menstruating women demonstrated the effect of training clearly (*p* < 0.05). Before and after the trainings, the CC risk factors mostly known by teachers were determined as: not following hygienic rules, smoking and sexually transmitted disease of the partner. The most commonly stated risk factors by women in studies conducted in various populations about the subject were: more than one sexual partner, onset of sexual relationship at an earlier age, presence of HPV virus, intra-uterine development and heredity [42, 38]. Sexual behaviors such as the onset of sexual relationship at an earlier age are not widely accepted within the culture of the Turkish society. Cultural characteristics can be suggested to be effective on the inability to express this risk factor by women. Within the cervical cancer early diagnosis practices, the knowledge of the teachers about HPV vaccine and PST application intervals were determined to be low, both before and after the trainings. Furthermore, in national and international studies conducted on the subject, it is deduced that women have very little knowledge regarding the application periods of the HPV vaccine and the PST [23, 32, 43]. Women in advanced age and those who are of higher education, those married and with children were reported to have higher knowledge levels about cervical cancer in previous studies [21, 23, 43]. However, in this study, it was expected from women to have higher knowledge levels regarding cervical cancer before the training, since they were teachers; the low knowledge levels show that even this group is not informed sufficiently. High levels of effect size were determined in terms of the teacher’s knowledge levels regarding cervical cancer risk factors and early diagnosis (2.11) and this result proved the positive effect of training.

It is thought that acquiring knowledge regarding all risk factors by the given training increases the awareness of the teachers on the prevention of breast and cervical cancer and this will be reflected positively in their behaviors.

### BSE, MMG and PST behaviors of teachers

It attracts attention that in the study, half of the teachers did not perform BSE, none of them had undergone MMG, and approximately 2/3 of them had not undergone PST before the education. In the studies regarding the subject, a positive attitude towards realization of behavior is important for changing the behavior in a positive manner. In the teachers’ behavior who did not perform BSE, while forgetting and not knowing how to do it were determined as the most frequently stated reasons, in the behavior of those not undergoing PST, unwillingness to be tested and thinking the test as being unnecessary were reported as the primary reasons. These results indicate that the negative attitudes of teachers towards PST were higher than that towards BSE. The research is a follow-up study and this is important for being a reminder and increasing the motivation.

### Stages of change regarding BSE

Although the BSE performing rates of female teachers in Turkey is lower than that in other parts of the world (from 10.2% to 27.3%), almost half of the teachers in the study group performed BSE before the training and this rate was similar to other country samples [6, 7, 10, 12]. It has attracted attention that teachers who did not perform regular BSE before the training, but intended to do so (preparation stage) and those who switched to regular BSE performing behavior at the end of the training (action and maintenance stages) increased in number significantly. The trainings and follow ups regarding the subject have created positive changes on BSE performing behaviors of teachers. This change is also supported by the determination of a medium level training effect size (Table 4).

### Behaviors of undergoing MMG

Both in the world and in Turkey, 10.1%–47.0% of the women over 40 years of age are determined to have undergone MMG [10, 12, 14]. As for the study, none of the teachers who were 40 years of age and older underwent or even considered MMG before the training. It was determined that after the training, 33.3% of the teachers had undergone MMG at time 3 and %38.9 at time 4. Although the MMG rates of the teachers were lower than that of the other studies, there was an important change observed in this behavior after the program, since there was no mammography before the training, and this result is also supported by the effect size of the intervention.

### Stages of change regarding PST

While the PST rates of the female teachers are between 31.2%–38% in developing countries, it is determined to be between %11.8-%65.8 in Turkey [22–24, 33, 44]. However, the rate of women undergoing PST is equal to or above 80% in developed countries [17, 20, 45, 46]. The rate of teachers undergoing the Pap test before the training was much lower than that of the relevant literature. In the study, while almost half of the teachers were in the pre-suggesting stage before the training in terms of undergoing PST, the rate of the teachers at time 3 and time 4 in the suggesting and taking action stages was 45.9%. This positive change of the changing stages revealed the necessity of the information and campaigns regarding the subject.

In the literature, it has been reported that factors such as knowledge, age and educational level of the individuals are effective on early diagnosis behaviors regarding breast and cervical cancers [7, 13, 22, 34, 46]. When the study group was assessed in terms of these characteristics, it has been suggested that the mid-level “knowledge” of the teachers about the subject, the mean “age” being within the risky period for cancer, and their “educational level” being mostly at high school level, can be effective factors for changing the early diagnosis behavior positively.

### Stages of change results of the students regarding BSE, MMG and PST

After the knowledge transfer to the students transferred by teachers, it was determined that all of those over 40 years of age had undergone mammography, 2/3 of all the students had undergone PST and almost all of them had positive behavior change in terms of performing BSE. A positive effect on behavior change of the knowledge transfer from teachers to students was determined regarding BSE, MMG and PST.

#### Study limitations

One of the most important limitations of this study is the lack of studies regarding the construct validity of the data collection instruments. The semi-experimental study was limited by the use of convenience sampling. However, due to the fact that the method used and the statistics having been explained in detail, the results reaching a mid-high level of effect size, and the results of different studies having been included in the discussion section, it has been considered that the results of the study can be generalized.

## Conclusion

At the end of the “breast and cervical cancer awareness” assessed on the basis of the Transtheoretical Model, there has been progress in a positive direction in the teachers’ knowledge score towards an increase regarding the behaviors towards early diagnosis of breast and cervical cancer, carrying out BSE, undergoing MMG and undergoing PST. The knowledge transfer from teachers to students was also similarly effective. According to the results, the trainings for cancer and early diagnosis can be given to the teachers who are the role models for students and societies within the framework of theoretical models tested, and the validity and reliabilities have been proven. Appropriate trainings to their stages can be given to the teachers who are in different stages of change. Teachers can share the information about the subject with students registered in their courses to create awareness. The results of this study provide practitioners (eg, practice nurses or public health nurses) information on how to develop an effective structured training program for female teachers and their training group and community. There are 981 Public Training Centers in Turkey and 3.261 female teachers teach in these institutions. Up to date, approximately 3 million women have participated in these courses in order to receive training. Studies similar to this study can always be carried out in these centers providing trainings to adults. Generalization of the findings can be made through comparisons.

## Additional file

Additional file 1: Datasets supporting the conclusions of this article. (ZIP 2 mb)

## Abbreviations

BC: Breast cancer
BSE: Breast self-examination
CBE: Clinical breast examination
CC: Cervical cancer
MMG: Mammography
PST: Pap smear test
TTM: The Transtheoritical Model

## References

[1] World Health Organization: Breast cancer estimated incidence, mortality and prevalence worldwiden. http://globocan.iarc.fr/Pages/fact_sheets_cancer.aspx (2012). Accessed 14 Apr 2015.

[2] Turkey Ministry of Health: Turkey cancer statistics (2009). http://kanser.gov.tr/haberler/921-2009-t%C3%BCrkiye-kanser-istatistikleri-yay%C4%B1mland%C4%B1.html.Accessed 14 Apr 2015. (in Turkish).

[3] World Health Organization: Breast cancer, prevention and control. http://www.who.int/cancer/detection/breastcancer/en/index2.html. Accessed 26 Oct 2015.

[4] Mai V, Sullivan T, Chiarelli A (2009). Breast cancer screening program in Canada, successes and challenges. Salud Publica Mex.

[5] Isara AR, Ojedokun CI (2011). Knowledge of breast cancer and practice of breast self examination among female senior secondary school students in Abuja, Nigeria. J Prev Med Hyg.

[6] Faronbi J, Abolade J. Breast self examination practices among female secondary school teachers in a rural community in Oyo state, Nigeria. Open J Nurs. 2012; doi:10.4236/ojn.2012.22017.

[7] Parsa P, Kandiah M, Parsa N (2011). Factors associated with breast self-examination among Malaysian women teachers. EMHJ.

[8] Sarfo AL, Awush-Peasah D, Asamoah EA (2013). Knowledge, attitude, and practice of self-breast examination among female university students at Presbyterian university college, Ghana. Am J Res Commun.

[9] Swan J, Breen N, Coates RJ, Rimer BK, Lee NC. Progress in cancer screening practices in the United States. Results from the 2000 national health interview survey. Cancer. 2003; doi:10.1002/cncr.11208. 10.1002/cncr.11208 12627518

[10] Akpınar YY, Baykan Z, Naçar M, Gün I, Çetinkaya F (2011). Knowledge and practice of breast cancer screening among female health care professionals in Turkey. Asian Pacific J Cancer Prev.

[11] Demirkiran N, Akdolun B, Memiş S, Türk G, Özvurmaz S, Tunçyürek P (2007). How do nurses and teachers perform breast self-examination: are they reliable sources of information?. BMC Public Health.

[12] Nur N. Breast cancer knowledge and screening behaviors of the female teachers. Women Health. 2010; doi:10.1080/03630241003601087. 10.1080/03630241003601087 20349394

[13] Seçginli S, Nahcivan N (2006). Factors associated with breast cancer screening behaviors in a sample of Turkish women: a questionnaire survey. Int J Nurs Stud.

[14] Sadikoglu G, Ozcakir A, Dogan F, Gökgöz S, Bilgen N (2010). Mammography utilization among Turkish women. Asian Pac J Cancer Prev.

[15] SY S, Huang J, Ho CC, Liaw YP. Evidence for cervical cancer mortality with screening program in Taiwan, 1981–2010: age-period-cohort model. BMC Public Health. 2013; doi:10.1186/1471-2458-13-13. 10.1186/1471-2458-13-13 PMC3543840 23297757

[16] World Health Organization: Comprehensive cervical cancer prevention and control: a healthier future for girls and women (2013). http://www.who.int/immunization/hpv/learn/comprehensive_cervical_cancer_who_2013.pdf. Accessed 27 Oct 2015.

[17] Gakidou E, Nordhagen S, Obermeyer Z. Coverage of cervical cancer screening in 57 countries: low average levels and large inequalities. PLoS Med. 2008; doi:10.1371/journal.pmed.0050132. 10.1371/journal.pmed.0050132 PMC2429949 18563963

[18] Harry TK, Felicia MS, Ngugen SA (2006). Needs assessment of barriers to cervical cancer screening in Vietnamese American health care providers. Californian J. Health Promotion.

[19] Rydström C, Törnberg S (2006). Cervical cancer incidence and mortality in the best and worst of worlds. Scand J Public Health.

[20] Sirovich BE, Welch HG (2004). The frequency of pap smear screening in the United States. J Gen Intern Med.

[21] Al-Meer FM, Aseel MT, Al-Khalaf J, Al-Kuwari MG, Ismail MF (2011). Knowledge, attitude and practices regarding cervical cancer and screening among women visiting primary health care in Qatar. East Mediterr Health J.

[22] Erbil N, Tezcan Y, Gür NE, Yıldırım M, Aliş N (2010). Factors affecting cervical screening among Turkish women. Asian Pacific. J Cancer Prev.

[23] Uysal A, Birsel A (2009). Knowledge about cervical cancer risk factors and pap testing behavior among Turkish women. Asian Pacific. J Cancer Prev..

[24] Esin MN, Bulduk S, Ardic A. Beliefs about cervical cancer screening among Turkish married women. J Cancer Educ. 2011; doi:10.1007/s13187-011-0198-y. 10.1007/s13187-011-0198-y 21336699

[25] Cancer Early Detection Education Center: The pap test screening in Turkey (2014). http://kanser.gov.tr/Dosya/Bilgi-Dokumanlari/ketem-el-kitabi.pdf. Turkish. Accessed 27 Oct 2015. (in Turkish).

[26] Rakowski W, Ehrich B, Dubé CE, Pearlman DN, Goldstein MG, Peterson KK, et al. Screening mammography and constructs from the transtheoretical model: associations using two definitions of the stages-of-adoption. Ann Behav Med. 1996; doi:10.1007/BF02909581. 10.1007/BF02909581 24203691

[27] Entisar AE, Om Ebrahiem AEE (2012). Applying the transtheoretical model of change and the health belief model to breast self-examination in females undergraduate students in Faculty of Nursing Tanta University. J Am Sci.

[28] Tung WC, Nguyen DH, Tran T. Applying the transtheoretical model to cervical cancer screening in Vietnamese-American women. Int Nurs Rev. 2008; doi:10.1111/j.1466-7657.2007.00602.x. 10.1111/j.1466-7657.2007.00602.x 18275539

[29] Lee JY, Hyeoun-Ae P, Yul HM. Transtheoretical model-based nursing intervention on lifestyle change: a review focused on intervention delivery methods. Asian Nurs Res. 2015; doi:10.1016/j.anr.2015.05.001. 10.1016/j.anr.2015.05.001 26160246

[30] TY W, West BT (2007). Mammography stage of adoption and decision balance among Asian Indian and Filipino American women. Cancer Nurs.

[31] Strong C, Liang W. Relationships between decisional balance and stage of adopting mammography and pap testing among Chinese American women. Cancer Epidemiol. 2009; doi:10.1016/j.canep.2009.10.002. 10.1016/j.canep.2009.10.002 PMC2787690 19900848

[32] Bala D, Gameti H (2011). An educational intervention study of breast self examination (BSE) in 250 women beneficiaries of urban health centers of west zone of Ahmedabad. Healthline.

[33] Yousof SA (2010). Breast cancer awareness among Saudi nursing students department of nursing, faculty of applied medical sciences king Abdulaziz university, Jeddah, Saudi Arabia. JKAU: Med Sci.

[34] Ekaete AT, Okeowo P (2014). Breast self examination among secondary school teachers in south-south, Nigeria: a survey of perception and practice. J Public Health Epidemiol.

[35] Kayode FO, Akande TM, Osagbemi GK (2005). Knowledge, attitude and practice of breast self examination among female secondary school teachers IIlorin, Nigeria. European. J Sci Res.

[36] Geray C (2002). Public education.

[37] Machin D, Campbell MJ, Tan SB, Tan SH (2007). Sample size tables for clinical studies.

[38] Esin N, Erdoğan S, Nahcivan N, Esin N (2014). Data collect methods and tools & validity and reliability of the data collect tools. Research in nursing, process, practice and critique.

[39] Polit DF, Beck CT, Nursing research: generating and assessing evidence for nursing practice. 9th ed. China:Wolters Kluwer Health;2012.

[40] Prochaska JO, DiClimente CC (1983). Stage and processes of self change of smoking: toward an integrative model of change. J Consult Clin Psychol.

[41] Cohen J (1988). Statistical power analysis for the behavioral sciences.

[42] Ertem G (2009). Awareness of cervical cancer risk factors and screening behavior among nurses in rural Turkey. Asian Pac J Cancer Prev.

[43] Shekhar S, Sharma C, Thakur S, Raina N (2013). Cervical cancer screening: knowledge, attitude and practices among nursing staff in a tertiary level teaching institution of rural India. Asian Pac J Cancer Prev.

[44] Sirin A, Atan S, Tasci E (2006). Protection from cancer and early diagnosis applications in Izmir, Turkey. Cancer Nurs.

[45] Tshering D, Pandup T. Cervical cancer knowledge and screening behaviors among female university graduates of year 2012 attending national graduate orientation program, Bhutan. BMC Womens Health. 2014; 10.1186/1472-6874-14-44. 10.1186/1472-6874-14-44 PMC3975232 24618416

[46] Neeraja BP, Murff LH, Yong C, Hargreaves M, Fowke JH. Papanicolaou testing among women in the southern United States. J Women's Health (Larchmt). 2008; doi:10.1089/jwh.2007.0576. 10.1089/jwh.2007.0576 PMC2942751 18582173

