# Supplementary figures and images for: Effect of structured training programme on the knowledge and behaviors of breast and cervical cancer screening among the female teachers in Turkey
Source: BMC Womens Health. 2017 Dec 7;17:123. doi: 10.1186/s12905-017-0478-8 (PMC5721587; doi:10.1186/s12905-017-0478-8)

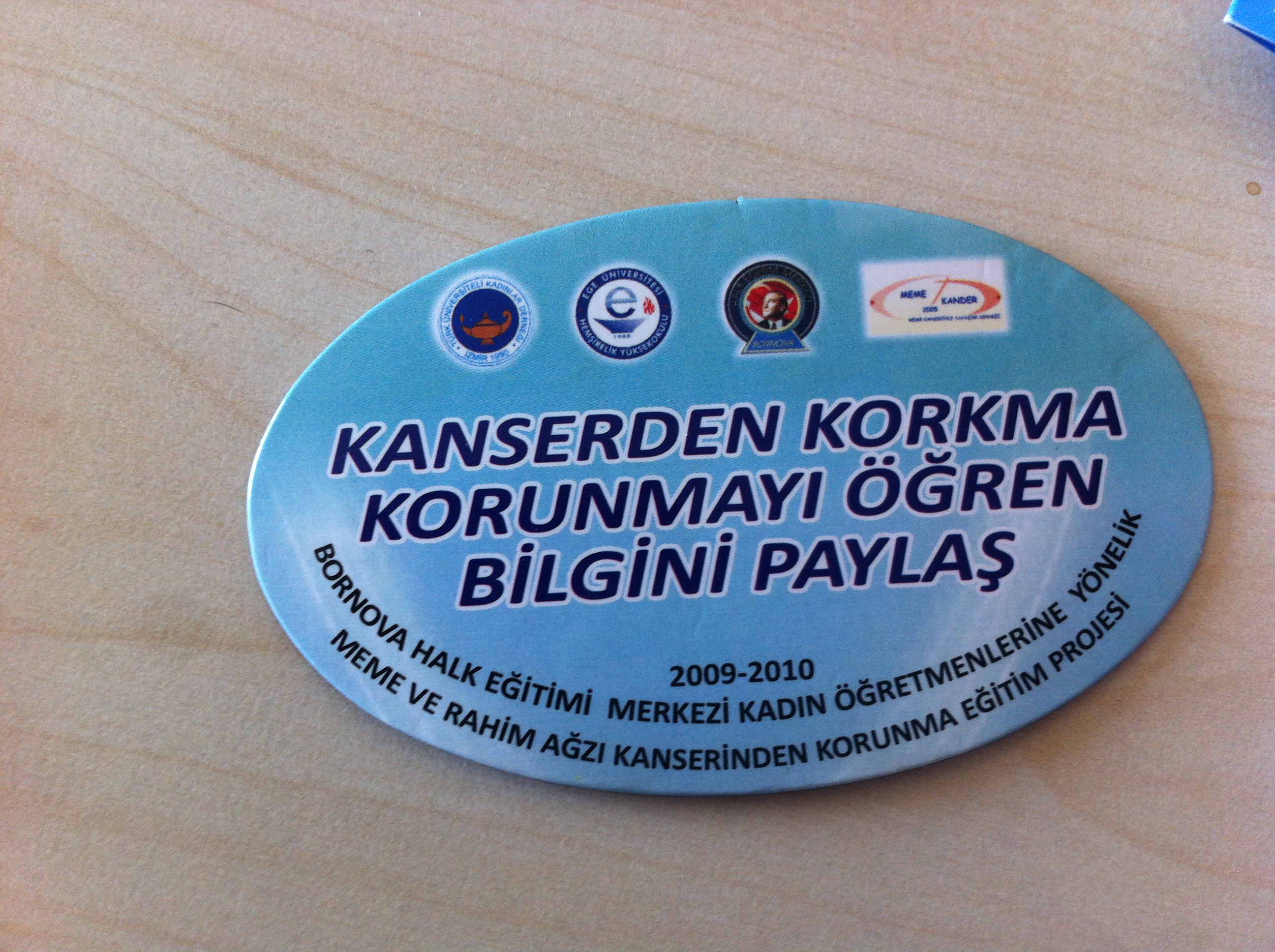

Supplement: Additional file 1: — Datasets supporting the conclusions of this article. (ZIP 2 mb) [file 12905_2017_478_MOESM1_ESM.zip › magnet.JPG]
